# Pulses of anthropogenic food availability appear to benefit parents, but compromise nestling growth in urban red-winged starlings

**DOI:** 10.1007/s00442-021-05033-3

**Published:** 2021-09-18

**Authors:** Sarah Catto, Petra Sumasgutner, Arjun Amar, Robert L. Thomson, Susan J. Cunningham

**Affiliations:** 1 FitzPatrick Institute of African Ornithology, DSI-NRF Centre of Excellence, University of Cape Town, Private Bag X3, Rondebosch, 7701 South Africa; 2 Konrad Lorenz Research Center, Core Facility for Behaviour and Cognition, Department of Behavioral and Cognitive Biology, University of Vienna, Grünau/Almtal, 4645 Austria

**Keywords:** Urbanisation, Global change, Nestling development, Food fluctuations, Body mass

## Abstract

The provision of anthropogenic food undoubtedly influences urban bird fitness. However, the nature of the impact is unclear, with both benefits and costs of urban diets documented. Moreover, the influence of short-term fluctuations in food availability, linked to urban weekday/weekend cycles of human presence, is largely unknown. We explored whether breeding red-winged starlings *Onychognathus morio* in Cape Town, South Africa, altered foraging and provisioning behaviour between days with high human presence (HHP) and days with low human presence (LHP)—i.e. weekdays versus weekends and vacation days. We investigated the relationship between starling diet, adult body mass and nestling development. Breeding adults consumed and provisioned the same quantity of food, but a significantly greater proportion of anthropogenic food on HHP compared to LHP days. Adults apparently benefited from the anthropogenic diet, experiencing significantly greater mass gain on HHP days. However, nestlings experienced a cost, with the number of HHP days during the nestling period associated negatively with nestling size. Adults may, therefore, benefit from the high calorie content of anthropogenic food, while nestlings may be negatively affected by nutrient limitation. The quantity of food available in urban environments may, therefore, benefit adult survival, while its quality imposes a cost to nestling growth.

## Introduction

Urbanisation dramatically alters the landscape, often leading to a complete restructuring of habitats and species composition and presenting organisms with multiple novel challenges (Shochat et al. 2006). Urban ecologists have documented numerous biotic and abiotic consequences of urbanisation, such as altered micro-climate (Collins et al. 2000), increased pollution (Kempenaers et al. 2010; Tsipoura et al. 2011; Slabbekoorn 2013), changes in species interactions and community composition (McKinney 2006), and changes in the abundance and types of food resources over both long (Luniak 2004) and short (Stofberg et al. 2019) timescales. The altered availability of food resources and the exploitation of anthropogenic food, in particular, are likely to have profound effects on the ecology of wildlife in cities (Robb et al. 2008a), with both positive and negative impacts on the fitness of individuals (Chamberlain et al. 2009; Stofberg et al. 2019). Indeed, the ability to successfully exploit novel urban food resources is likely a major component underlying the success (or otherwise) of species in adapting to and exploiting urban environments (Chace and Walsh 2006; Kark et al. 2007).

In birds, evidence regarding the fitness impacts of an anthropogenic diet is equivocal. Positive impacts of anthropogenic food subsidies include increased population sizes and improved body condition during winter that can carry over into greater reproductive success in the subsequent breeding season (Robb et al. 2008b). However, studies comparing productivity of urban and non-urban birds have found emergent patterns suggesting that anthropogenic food can have both positive and negative effects on birds’ demographic parameters, even within the same population. For example, urbanisation and high availability of anthropogenic food is associated with advanced laying dates but reduced brood sizes in Blue and Great Tits (*Cyanistes caeruleus, Parus major*) (Harrison et al. 2010), and increased nestling provisioning rates, but reduced nestling mass in House Wrens (*Troglodytes aedon*) (Newhouse et al. 2008). This suggests that positive effects on the body condition of adults allowing them to lay early, for example, may not be reflected in better breeding outcomes.

This diversity of impacts of anthropogenic diets on bird fitness and demographics is puzzling but may be related to food quality (Seress and Liker 2015). Anthropogenic food discards tend to have high calorific content but are also high in carbohydrates and low in proteins (Seress and Liker 2015). Increased overwinter survival and body condition of adult birds associated with anthropogenic food subsidies may indicate that adults are calorie rather than nutrient limited. However, poor nutritional quality (Andersson et al. 2015; Isaksson et al. 2017) of anthropogenic food items and limited availability of natural food items in urban areas (Plummer et al. 2019) may result in suppressed breeding success due to macronutrient limitation affecting egg production and nestling development (Coogan et al. 2018). Furthermore, natural food items in an urban setting might be of lower quality. For example, the urban climate affects carotenoid-synthesizing trees (Isaksson 2009), which restricts the carotenoid content of the entire urban food chain including caterpillars (Isaksson and Andersson 2007)—a staple diet for many nestlings. Carotenoids are important antioxidants, and their limitation can have negative health impacts (e.g.Isaksson et al. 2005; Sumasgutner et al. 2018). Additionally, caterpillar availability in urban areas is reduced compared to rural areas, as seen in and around Glasgow, Scotland, where urban Blue Tit parents consequently provisioned fewer caterpillars to their offspring than rural conspecifics, negatively impacting fledging success (Pollock et al. 2017). Similarly, Common Blackbird (*Turdus merula*) nestlings in urban areas display significantly higher starvation rates than those in woodland areas (Ibáñez-Álamo and Soler 2010). This suggests that, for some urban bird populations, appropriate food items for nestlings are in poor supply and anthropogenic alternatives are inappropriate substitutes.

In addition to changes in food quality, large-scale fluctuations in anthropogenic food availability also occur in urban areas. Although food might be more predictably available over seasonal and annual timescales in urban than rural environments (Fuller et al. 2019), the same might not be true over shorter periods of days and weeks. A typical urban regime is that of pulsed availability of anthropogenic food on weekly cycles, especially in areas such as central business districts and educational institutions which see much larger numbers of people during weekday working hours than on weekends and during vacation periods (Stofberg et al. 2019; Spelt et al. 2021). To date, hardly any literature exists on how these well-pronounced short-term fluctuations might affect urban birds. Weekly cycles of food availability appear to benefit body mass maintenance in non-breeding red-winged starlings (*Onychognathus morio*) in Cape Town on weekdays versus weekends (Stofberg et al. 2019), but further data are sparse on impacts of short-term cyclic fluctuations in anthropogenic food on survival, breeding success or productivity of urban birds globally.

Here, we took advantage of a ‘natural experiment’ occurring on the University of Cape Town campus in South Africa, where a breeding population of urban-exploiting red-winged starlings (hereafter ‘starlings’) are exposed to fluctuating amounts of anthropogenic food over short temporal scales, to address the question of whether adult and nestling starlings are differentially affected by diets high in anthropogenic food. During the week, on high human presence (hereafter ‘HHP’) days, thousands of students and an associated high abundance of anthropogenic food provide heavily pulsed foraging opportunities for the resident starling population, with this food supply withdrawn over weekends and vacations (‘low human presence’ or ‘LHP’ days), forcing starlings to switch to a more natural diet including more insects and berries (Stofberg et al. 2019; Risi et al. 2021). We hypothesised that adult starlings would benefit from readily available high-calorie food on HHP days; whereas nestlings would be negatively impacted as a result of being fed a high proportion of low-quality anthropogenic food. We, therefore, predicted that breeding adults would eat a higher proportion of anthropogenic food and gain more body mass between morning and evening on HHP than on LHP days. We also predicted starlings would feed a higher proportion of anthropogenic food to their nestlings on HHP days than LHP days. If nestling development in urban birds is affected by nutrient limitation associated with an anthropogenic diet, we expected that nestlings experiencing a high proportion of HHP days between hatch and fledge would be smaller and lighter than those experiencing a high proportion of LHP days.

## Materials and methods

### Study site and species

This study took place on the upper campus of the University of Cape Town (hereafter ‘UCT’; 33° 57′ 27.5" S, 18° 27′ 40.31" E), Western Cape, South Africa. Over 25,000 students are enrolled at UCT annually and, on weekdays during the academic term, most of these students attend classes on upper campus. This abundance of people corresponds with an abundance of anthropogenic food provided by cafeteria stalls and brought by students from off campus (Stofberg et al. 2019; Risi et al. 2021). Cafeteria stalls are closed during weekends and the number of students visiting campus is dramatically reduced—e.g. daily admission records from the upper campus library are on average twice as high on weekdays (x̄ = 2892 students, CI [2175; 3610] students) than weekends (x̄ = 1489 students, CI [962; 2017] students) (two-sample *t* test, *t* = − 2.8776, df = 45, *P* = 0.006; see detailed statistics in Stofberg et al. (2019)).

Red-winged starlings *(Onychognathus morio)* are common, medium-sized (115–155 g) omnivorous birds with a native range from Ethiopia to the Cape in South Africa (Craig 2005). They are described as a gregarious bird, but resident pairs appear together throughout the year. Laying dates are between September and March and, although they are primarily a cliff-nesting species, starlings have been documented breeding on buildings at UCT since the 1940s (Rowan 1955). While their natural diet consists of fruit, nectar, seeds and arthropods, UCT’s resident population is often seen scavenging anthropogenic food dropped by students and retrieving it from rubbish bins (du Plessis 2005).

### Monitored nests and pairs

Between Apr-2017 and Aug-2017, ~ 130 adult starlings were captured on UCT’s upper campus using spring traps baited with raisins or processed cheese. Each starling was individually marked with three colour rings and one metal SAFRING band with a unique alphanumeric sequence.

We conducted nest watches and behavioural observations of 15 breeding pairs with at least one colour-ringed partner between 5-Nov-2017 and 11-Dec-2017. Behaviour and nest watch data were collected every Friday, Saturday, Sunday and Monday to facilitate direct comparisons between weekdays (hereafter ‘high human presence’ or ‘HHP’ days) and weekends (hereafter ‘low human presence’ or ‘LHP’ days). Each day of data collection was divided into three time-blocks, each containing two 1-h observation slots: morning: 08:30–09:30, 10:00–11:00; early afternoon: 12:00–13:00, 13:30–14:30; and late afternoon: 15:30–16:30, 17:00–18:00. During each 1-h observation slot, we monitored one breeding starling pair (‘behavioural observations’, below) and their nest (‘nest watches’, below) in parallel.

### Behavioural observations: adult diet and behaviour

During each 1-h slot, we performed two ~ 20-min-long behavioural focal observations (hereafter ‘focals’)—one on each member of the starling pair associated with the nest being monitored. This involved following an individual at a distance of 2–3 m (possible due to the habituation of the birds to the heavy human presence on campus) and recording behaviour using CyberTracker software (The CyberTracker Team 1997) (http://www.cybertracker.org/): a customisable data collection app loaded onto a smartphone. When a starling flew out of sight, the duration of absence was recorded and subsequently removed from all analyses.

Birds were recorded as ‘foraging’ when visually searching for and handling food items. Items were recorded as ‘swallowed’ if this action was seen, or ‘loaded’ if the bird held the item in its beak and flew towards the nest—usually a precursor to provisioning nestlings. We used ‘beakful’ as a unit to quantify the food amount rather than ‘item’ as some items were larger than others. In addition to the number of beakfuls loaded or swallowed, we recorded whether the item was of anthropogenic (e.g. bread, noodle, apple) or natural (e.g. insect, berry, seed) origin. All focals were performed by the lead author to avoid introducing observer effects.

### Nest watches: nestling diet

One-hour-long nest watches were performed concurrently with the focals described above. Trained volunteers recorded all activity at the nest for the full hour using CyberTracker software. We used these data to determine: (1) provisioning rates to nestlings; and (2) the proportion of provisioned food that was of anthropogenic origin.

### Nestling mass and developmental parameters

We ringed and collected morphometric data from 12 nestlings across eight nests in 2017 and nine nestlings across six nests in 2018 at approximately day 16 (ranged 16–18) after hatching as red-winged starling nestlings are fully feathered by this time, but the risk of forced fledging is low with a nestling period of 22–28 days (Craig 2005). Measurements taken included: (1) mass (g); (2) head length (mm); (3) tarsus length (mm); and (4) wing length (mm). These parameters were included in a principal components analysis (see statistical analyses below), and explored in relation to the number of HHP days experienced by nestlings in the 15 days prior to ringing, to investigate nestling size in relation to anthropogenic food availability.

### Adult daily mass change

To measure adult body mass change between the morning and evening, colour-ringed starlings were trained to stand on a portable top-pan scale in return for a small food reward (a raisin) twice per day (morning and evening) following Stofberg et al. (2019) and Ridley and Raihani (2007). As there was some variation in the exact time at which mass measurements could be made, the daily proportional mass change per individual was calculated and standardised to a 12-h day using the equation from du Plessis et al. (2012), as follows:

$${\text{Percentage mass change}} = \left( {\left( {w2{-}w1} \right)/w1} \right)/\left( {\left( {t2 - t1} \right)/12} \right) \times 100,$$

where *w*1 = morning mass, *w*2 = evening mass, *t*1 = time at which morning mass was taken, *t*2 = time at which evening mass was taken.

### Statistical analyses

All analyses were conducted in the R statistical environment (v. 3.3.2 and 3.5.0) (R Core Development Team 2016). Linear and Generalised Linear Mixed Models (LMMS and GLMMs) were implemented using the packages *lme4* (Bates et al. 2015) (normally distributed data) and *glmmADMB* (Skaug et al. 2016) (all other data distributions). Binomial, Poisson and negative binomial models were checked for overdispersion. For LMMs, the assumption of normality of residuals was checked by visually inspecting residual plots. For all analyses, we used an information theoretic approach and model averaging with the package *MuMIn* (Bartoń 2013). In each case, a candidate model set was generated with the same response variable, but always considering the key fixed predictor variable day status (categorical with two levels, HHP and LHP days) with different fixed covariates that could affect the response variable, as listed below for each analysis (e.g. sex of focal bird, time of day at which data were collected, nestling age and brood size). All candidate model sets contained the global model with the day status predictor and all relevant covariates and random intercepts, the null model (random intercepts only), and a set of candidate models always including the key day status predictor term and all possible nested combinations of the fixed covariates appropriate to that analysis. Our data set contained no missing values, ensuring accurate model comparisons throughout the selection and, if applicable, averaging process. The model set was then ranked using ΔAICc values. Akaike weights (*ω*i) were calculated to assess the relative likelihood for each model considered (Burnham and Anderson 2002). Thus, *ω*i reflected model probability given the full model set rather than only those below a given threshold of ΔAICc. A table of best candidate models (up to ΔAICc < 2.0) was extracted and used for model averaging (Anderson et al. 2001). We report the direction of parameter estimates and their magnitudes (effect sizes), and adjusted SEs and CIs (95% confidence limit) from model-averaged coefficients. We report adjusted SE because this incorporates model selection uncertainty, as opposed to standard SE which only considers sampling variance (Grueber et al. 2011). We used confidence intervals to assess the magnitude of the effect and concluded that the estimate is different from zero (i.e. there is a significant effect) when the confidence interval excludes zero. We decided on a model selection procedure based on the principles of parsimony (the largest amount of variance explained with the minimum number of predictors (Burnham and Anderson 2002)) to ensure that models are not overfitted with too many predictors given our sample size limitations while still accounting for potential effects of covariates such as sex, chick age and the time of data collection.

### Adult diet and foraging behaviour based on focal data

For breeding adults, we modelled: (1) the proportion of anthropogenic versus natural food consumed; (2) foraging effort (i.e. proportion of focal spent foraging versus not foraging) using the cbind syntax in *glmmADMB* (Skaug et al. 2016) with a binomial family and logit function; and, (3) food intake rate (i.e. total amount of beakfuls consumed) by fitting a negative binomial error structure (family = nbinom1; variance = ϕµ) in *glmmADMB* (Skaug et al. 2016) without zero inflation and the log of focal length (in minutes) as offset variable. For all candidate model sets, the global model included the fixed predictor day status (categorical with two levels, HHP or LHP), and fixed covariates time of day (categorical with three levels, morning, early afternoon or late afternoon), sex of adult (categorical with two levels, female or male) and age of nestlings (categorical with three levels, week 1, week 2, week 3). Bird ID nested within Nest ID was fitted as a random intercept in each model to incorporate the dependency among observations of the same individuals from the same nests, along with Week ID (a unique identifier for each week of the breeding season, included to account for seasonal effects).

### Nestling diet based on nest watch data

For data analysed at nest level, adult sex was not included as a covariate in the analyses because the variables of interest were nestling diet composition and food provisioning rates, with contributions of both parents summed. We modelled: (1) the proportion of nestlings’ diet that was made up of anthropogenic food versus natural food using the cbind syntax in *glmmADMB* (Skaug et al. 2016) with a binomial family and logit function; and (2) provisioning rate using zero-inflated Poisson error structure and log-link function in *glmmADMB* (Skaug et al. 2016). For both analyses, global models included the fixed predictor day status, and fixed covariates time of day and age of nestlings with Nest ID and Week ID as random intercepts.

### Nestling mass and morphometric data

To test predictions concerning the relationships between the number of HHP days nestlings experienced and their size at ringing age, we first conducted a principal components analysis using the function *princomp* (R Core Development Team 2016) on scaled ringing-age mass, head length, wing length and tarsus length to extract a combined variable describing nestling size. The first principal component (PC1) explained 74% of the variation in the data, and was positively loaded by all four morphometric variables (PC1 loadings: body mass: 0.52; head length: 0.43; tarsus length: 0.55; wing length: 0.49), such that higher PC1 scores indicate larger nestlings. We then modelled PC1 using LMMs with Gaussian error structure and identity-link functions in *lme4* (Bates et al. 2015)*.* The global model for this analysis included the fixed predictor HHP days (continuous, the number of HHP days during the nestling period), and fixed covariates nestling age at ringing (continuous, ranging from 16 to 18 days), brood size (continuous, ranging from 1 to 3) and year (categorical with two levels, 2017 or 2018), and Nest ID as a random intercept, to incorporate the dependency among individuals from the same nests.

### Adult mass data

To test for differences in daily percentage mass change in breeding adults between HHP and LHP days, we fitted LMMs with Gaussian error structure and identity-link functions in *lme4* (Bates et al. 2015). The global model included fixed predictor day status (categorical with two levels, HHP and LHP) and fixed covariate sex of adults (categorical with two levels, female and male), and Bird ID nested in Nest ID as random intercept, to incorporate the dependency among observations of the same individuals from the same nests.

## Results

Between 5 November and 11 December (22 days of data collection), we collected 35.9 h of focal data (163 individual focals) from 30 individual colour-ringed starlings in 15 breeding pairs (15 males and 15 females), across 15 nesting attempts. The number of focal observations performed per individual ranged from 3 to 9, with an average number of 5 ± 0.1. Focals were performed for 20 min, with an average of 12.2 ± 0.6 min of data collected per focal (time the birds spent out of sight was excluded from analyses). We concurrently performed a total of 85 1-h nest watches on the 15 nests of the same pairs. The number of nest watches performed ranged from 3 to 9 per nest, with an average of 6 ± 0.2 watches per nest. No nests were observed more than once on any single day.

### Adult diet and behaviour

**Fig. 1:**
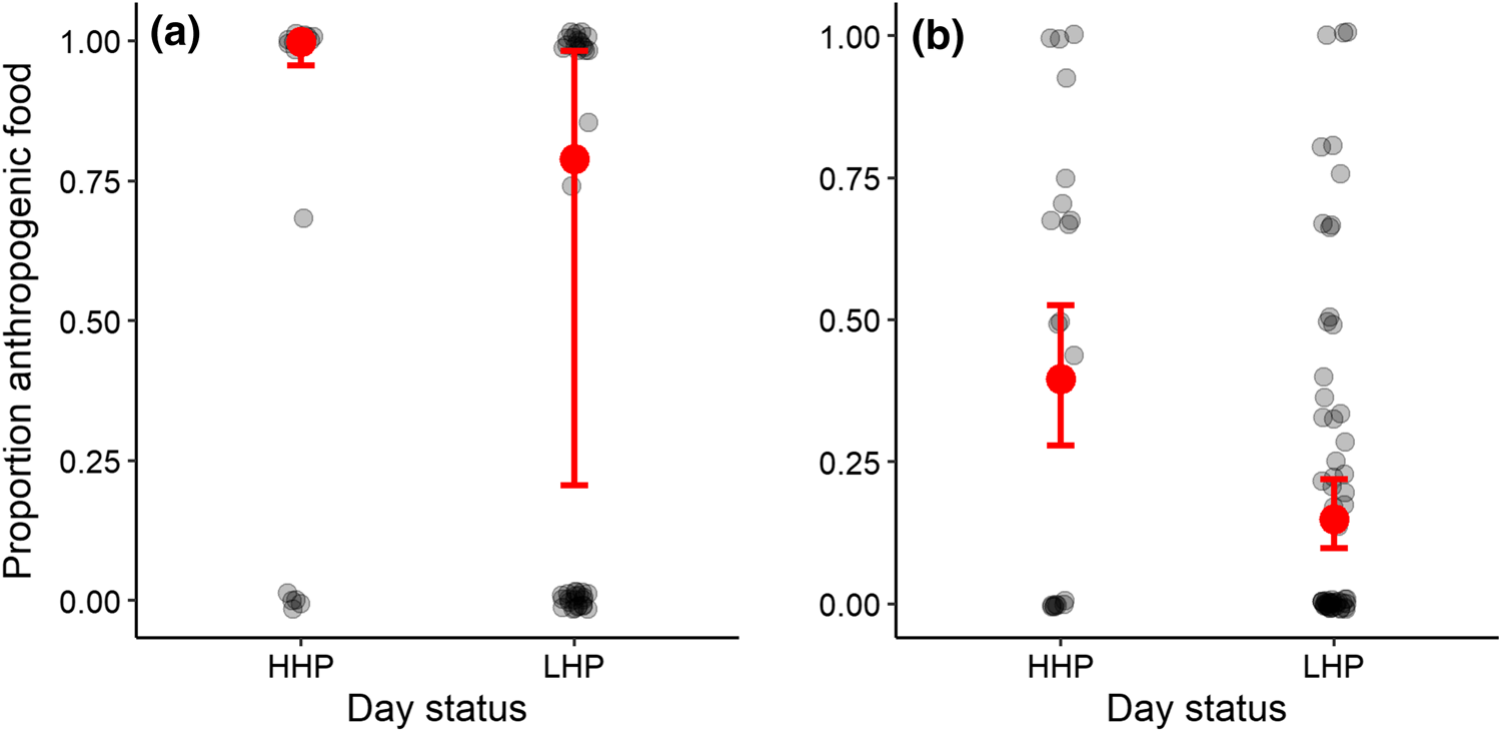
The proportion of anthropogenic food in the diet of **a** adult breeding red-winged starlings; and **b** nestlings on high human presence (HHP) and low human presence (LHP) days at the University of Cape Town. Adults consumed a greater proportion of anthropogenic food on HHP days (model-averaged mean = 0.99 [0.96, 1.00]) than LHP days (model-averaged mean = 0.79 [0.21, 0.98]). Nestlings were fed a greater proportion of anthropogenic food on HHP days (model-averaged mean = 0.40 [0.28, 0.52]) than LHP days (model-averaged mean = 0.15 [0.10, 0.22]). Data collected from 60 focal observations (27 HHP focals and 33 LHP focals) and 82 nest watches (22 HHP nest watches and 60 LHP nest watches). Error bars represent one standard error around the mean

Adult starlings consumed at least one food item during 60 of the 163 focals (36.8%; 27 HHP focals and 33 LHP focals, respectively). During these focals, adults consumed a greater proportion of anthropogenic food on HHP days (back-transformed model-averaged mean = 0.99 [0.96, 1.00]) than LHP days (back-transformed model-averaged mean = 0.79 [0.21, 0.98]) (Table 1a; Fig. 1a). However, the time they spent foraging per focal and their overall food intake was similar on both HHP and LHP days (Table 1b, c).

**Table 1:** Adult starling **a** diet (binomial); **b** foraging effort (binomial); and **c** food intake rate (negative binomial GLMMs with log-link function) in relation to day status (high human presence, HHP; or low human presence, LHP), time block (morning, early afternoon or late afternoon), sex of adult (male or female) and age of nestlings (weeks one, two or three)

(a) Response: adult diet (proportion anthropogenic food consumed) (*n* = 60 observations of 22 birds from 14 nests)				
		AICc	ΔAICc	ώ_i_
Top model set				
Day status + Time block + Sex		143.6	0.00	0.519
Day status + Time block		144.6	1.04	0.309

	Estimate	Adj. SE	2.5%	97.5%
Effect size of explanatory terms				
Intercept	**6.27**	**3.20**	**0.01**	**12.55**
Day status^Ϯ^ ‘LHP’	**− 4.95**	**1.70**	**− 8.29**	**− 1.62**
Time block^†^ ‘late afternoon’	**− **0.31	1.35	**− **2.96	2.34
Time block^†^ ‘morning’	**− 4.79**	**1.87**	**− 8.45**	**− 1.13**
Sex^ϰ^ ‘male’	**− **1.83	1.98	**− **6.37	0.51

(b) Response: foraging effort (proportion time spent foraging) (*n* = 163 observations of 30 birds from 15 nests)				
		AICc	ΔAICc	ώ_i_
Top model set				
Day status + Sex		480.0	0.00	0.595

	Estimate	SE	2.5%	97.5%
Effect size of explanatory terms				
Intercept	**− 2.50**	**0.17**	**− 2.83**	**− 2.17**
Day status^Ϯ^ ‘LHP’	**− **0.10	0.17	**− **0.44	0.24
Sex^ϰ^ ‘male’	**0.43**	**0.16**	**0.11**	**0.74**

(c) Response: food intake rate (beakfuls) (*n* = 163 observations of 30 birds from 15 nests)				
		AICc	ΔAICc	ώ_i_
Top model set				
Null model		478.1	0.00	0.574

	Estimate	SE	2.5%	97.5%
Effect size of explanatory terms				
Intercept	**− 2.11**	**0.23**	**− 2.56**	**− 1.66**

AICc, ΔAICc and model weights (*ω*i) are presented for all models within Δ2AICc for each analysis. Estimates, standard errors and 95% confidence limits presented are for the top model in the case where only one model within Δ2AICc was returned, or are model-averaged coefficients in the case of more than one competing model within Δ2AICc (in which case adjusted SEs are presented). Estimates are not back-transformed. Factors highlighted in bold have confidence intervals which do not contain zero

Ϯ Day status: ‘HHP’, † time block: ‘early afternoon’, ϰ sex: ‘female’; and ǂ age: ‘week one’ were used as reference categories

### Nestling diet

Nestlings were provisioned at least one food item in 82 of the 85 1-h nest watches we performed (96.5%; 22 HHP nest watches and 60 LHP nest watches, respectively). They were fed a greater proportion of anthropogenic food on HHP days (back-transformed model-averaged mean = 0.40 [0.28, 0.52]) than LHP days (back-transformed model-averaged mean = 0.15 [0.10, 0.22]) (Table 2a; Fig. 1b). However, the total number of beakfuls of food delivered to the nest was not influenced by day status (Table 2b).

**Table 2:** **a** Nestling diet (binomial); and **b** adult starling provisioning rates (zero-inflated Poisson GLMMs with log-link function) in relation to day status (high human presence, HHP; or low human presence, LHP), time block (morning, early afternoon or late afternoon) and age of nestlings (weeks one, two or three)

(a) Response: nestling diet (proportion anthropogenic food provisioned) (*n* = 82 nest watches from 15 nests)				
		AICc	ΔAICc	ώ_i_
Top model set				
Day status + Time block + Age		269.3	0.00	0.940

	Estimate	SE	2.5%	97.5%
Effect size of explanatory terms				
Intercept	− 0.42	0.53	− 1.46	0.62
Day status^Ϯ^ ‘LHP’	− **1.32**	**0.35**	− **2.00**	− **0.64**
Time block^†^ ‘late afternoon’	**0.72**	**0.33**	**0.06**	**1.37**
Time block^†^ ‘morning’	− **0.93**	**0.32**	− **1.56**	− **0.29**
Age^ǂ^ ‘week two’	0.74	0.38	− 0.02	1.49
Age^ǂ^ ‘week three’	− 0.29	0.47	− 1.21	0.63

(b) Response: provisioning rate (*n* = 85 nest watches from 15 nests)				
		AICc	ΔAICc	ώ_i_
Top model set				
Day status + Time block + Age		469.5	0.00	0.516
Day status + Time block		470.8	1.34	0.264

	Estimate	Adj. SE	2.5%	97.5%
Effect size of explanatory terms				
Intercept	**1.71**	**0.17**	**1.38**	**2.04**
Day status^Ϯ^ ‘LHP’	0.04	0.13	− 0.21	0.29
** Time block**^**†**^** ‘late afternoon’**	**− 0.38**	**0.14**	− **0.66**	− **0.10**
Time block^†^ ‘morning’	− 0.06	0.12	− 0.29	0.17
** Age**^**ǂ**^** ‘week two’**	**0.30**	**0.14**	**0.03**	**0.57**
Age^ǂ^ ‘week three’	0.08	0.16	− 0.22	0.39

AICc, ΔAICc and model weights (*ω*i) are presented for all models within Δ2AICc for each analysis. Estimates, standard errors and 95% confidence limits presented are for the top model in the case where only one model within Δ2AICc was returned, or are model-averaged coefficients in the case of more than one competing model within Δ2AICc (in which case adjusted SEs are presented). Estimates are not back-transformed. Factors highlighted in bold have confidence intervals which do not contain zero

Ϯ Day status: ‘HHP’, † time block: ‘early afternoon’, and ǂ age: ‘week one’ were used as reference categories

### Nestling size at ringing age

**Fig. 2:**
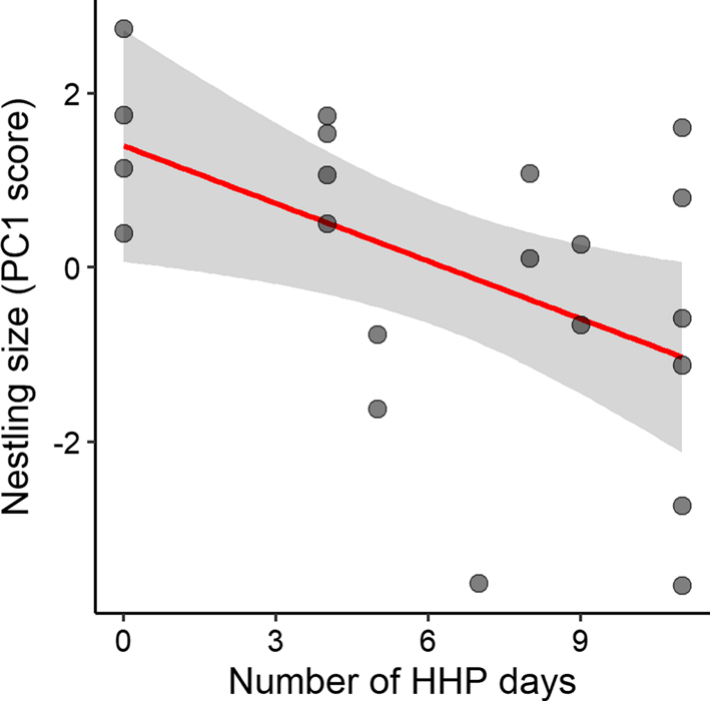
Red-winged starling nestling size decreases with increasing numbers of high human presence (HHP) days experienced in the 15 days prior to measurement. ‘Size’ is indicated by the first principal component (PC1) from a principal components analysis incorporating body mass, head, tarsus and wing length. PC1 explained 74% of the variation in the data and was positively loaded by all four morphometric variables (body mass: 0.52; head length: 0.43; tarsus length: 0.55; wing length: 0.49) such that higher PC1 scores indicate larger nestlings. *n* = 21 nestlings from 14 nests

We measured a total of 21 nestlings aged 16–18 days from 14 nests (13 nestlings in 2017 and 8 in 2018). Controlling for nestling age, increasing numbers of HHP days in the 15 days prior to measurement were associated with reduced nestling size (as indicated by PC1 scores; Table 3; Fig. 2).

**Table 3:** Linear mixed effects models with Gaussian error structure and identity-link function for ringing-age nestling size as indicated by the first principal component (PC1) from a principal components analysis incorporating body mass, head, tarsus and wing length

Response: PC1 (nestling size) (*n* = 21 nestlings from 14 nests)				
		AICc	ΔAICc	ώ_i_
Top model set				
HHP days + Age + Brood + Year		76.9	0.00	0.644
HHP days + Age + Year		78.3	1.39	0.321

	Estimate	Adj. SE	2.5%	97.5%
Effect size of explanatory terms				
Intercept	**− 23.33**	**5.76**	**− 34.63**	**− 12.03**
HHP days	**− 0.22**	**0.09**	**− 0.39**	**− 0.04**
Age	**1.54**	**0.34**	**0.88**	**2.20**
Brood	**− 1.15**	**0.47**	**− 2.08**	**− 0.23**
Year^Ϫ^ ‘2018’	**− **0.14	0.66	**− **1.43	1.14

PC1 explained 74% of the variation in the data and was positively loaded by all four morphometric variables (body mass: 0.52; head length: 0.43; tarsus length: 0.55; wing length: 0.49) such that higher PC1 scores indicate larger nestlings. AICc, ΔAICc and model weights (*ω*i) are presented for all models within Δ2AICc. Estimates, adjusted standard errors and 95% are model-averaged coefficients from the two competing top models. Factors highlighted in bold have confidence intervals which do not contain zero

Ϫ Year: ‘2017’ was used as the reference category

### Adult daily percentage mass change

**Fig. 3:**
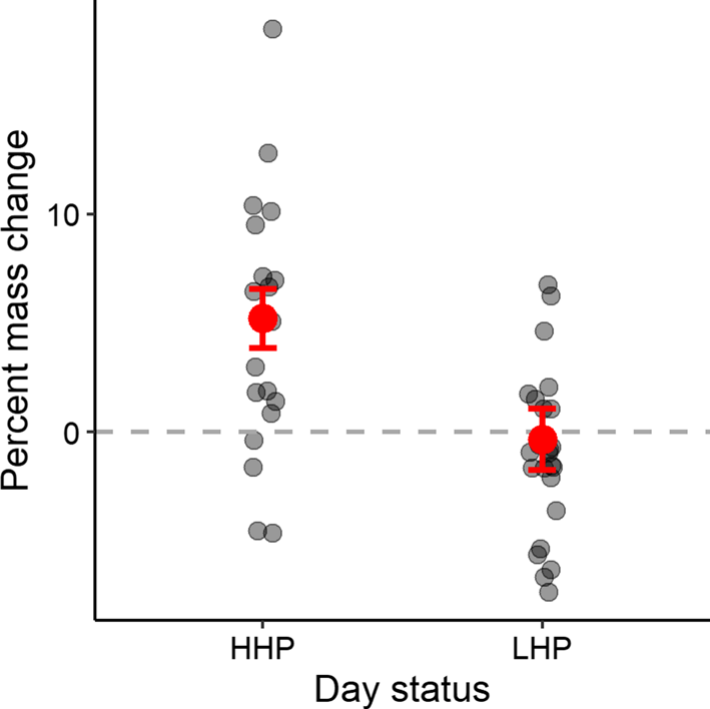
Adult red-winged starling daily % mass change on high human presence (HHP) and low human presence (LHP) days. Percentage mass change within the same colour-marked individuals was, on average, positive on HHP days (model-averaged mean = 5.22% ± 1.35%) but zero-to-negative on LHP days (model-averaged mean = − 0.37% ± 1.53%). Data collected on 20 HHP and 23 LHP days, respectively. *n* = 16 adult starlings from 9 nest attempts. Error bars represent one standard error around the mean

Paired morning and evening mass measurements from the same individual birds totalling 43 bird-days of mass data were obtained from 16 individuals within our study sample, including 9 males and 7 females. Adult percent mass change between morning and evening measurements was higher on HHP than LHP days (Table 4). Percentage mass change was, on average, positive on HHP days (model-averaged mean = 5.22% ± 1.35%), but zero-to-negative on LHP days (model-averaged mean = − 0.37% ± 1.53%) (Fig. 3).

**Table 4:** General linear mixed effects models with Gaussian error structure and identity-link function for adult starling daily percentage mass change (between morning and evening mass measurements on the same colour-marked individuals) in relation to day status (high human presence, HHP, or low human presence, LHP) and sex of adult (male or female)

Response: daily percentage mass change (*n* = 43 bird-days of data from 16 adult starlings from 9 nest attempts)				
		AICc	ΔAICc	ώ_i_
Top model set				
Day status + Sex		262.7	0.00	0.608
Day status		263.6	0.89	0.390

	Estimate	Adj. SE	2.5%	97.5%
Effect size of explanatory terms				
Intercept	**5.22**	**1.35**	**2.57**	**7.87**
Day status^Ϯ^ ‘LHP’	**− 5.54**	**1.58**	**− 8.63**	**− 2.45**
Sex^ϰ^ ‘male’	**− **1.46	1.58	**− **4.56	1.63

AICc, ΔAICc and model weights (*ω*i) are presented for all models within Δ2AICc. Model-averaged estimates, adjusted standard errors and 95% confidence limits are displayed. Factors highlighted in bold have confidence intervals which do not contain zero

Ϯ Day status: ‘HHP’ and ϰ sex: ‘female’ were used as reference categories

## Discussion

We investigated the impact of short-term (weekly) fluctuations in anthropogenic food availability on parental care and nestling condition of urban-exploiting red-winged starlings, and found that adult birds appeared to benefit from a high proportion of anthropogenic food in their diet on HHP days, whereas nestlings apparently suffered a cost associated with this diet. Breeding birds were able to maintain similar food intake rates and provisioning rates to nestlings regardless of human presence on (UCT’s upper) campus. However, the diet they consumed and the diet they provisioned to their nestlings shifted considerably with the type of day (i.e. weekdays during term time with high human presence *versus* weekends and vacation days with low human presence). Breeding starlings heavily exploited anthropogenic food sources on HHP days, when close to 100% of the adult starlings’ diet and approximately 40% of food provisioned to nestlings was of anthropogenic origin. They shifted their diet to include more natural food on LHP days, with only approximately 80% of adult diet and 15% of nestling diet of anthropogenic origin on these days. Adult birds gained mass on HHP days but maintained or lost mass on LHP days, while nestlings showed an opposite response: increased exposure to HHP days during the nestling period was correlated with overall smaller body size of nestlings at ringing age.

Starlings in our study appear to behaviourally adjust their foraging, switching dietary composition to take advantage of varying food availability and adjusting to human time schedules. Monitored birds incorporated a larger proportion of readily available anthropogenic food into their diet and that of their nestlings according to availability, resulting in adults gaining mass on HHP days without an associated increase in foraging effort or food intake rates. This is likely a result of the relatively high calorific content of anthropogenic food in comparison to natural food items (Auman et al. 2008; Coogan et al. 2018). This behaviour and its outcomes are considered important contributing factors to the successful colonisation of urban habitats by birds (Sol et al. 2013; Spelt et al. 2021), and have been described previously: Silver Gulls (*Larus novaehollandiae*) at an urbanised site with abundant anthropogenic food in Tasmania, Australia were heavier and had greater body condition than non-urban gulls (Auman et al. 2008). In our study system, however, we cannot rule out potential negative effects on the health of the birds that might be reflected in physiological parameters we did not measure, such as fatty acid profiles (Andersson et al. 2015) or cholesterol levels (Townsend et al. 2019). To determine whether adults truly are benefitting from anthropogenic food, further work could incorporate physiological measurements of the health of the birds, focusing on variables tightly linked to diet that change over short timescales.

While breeding adult starlings appear to be benefitting from the increased energy associated with anthropogenic food on HHP days, the available macronutrients in this diet appear to be negatively impacting nestling growth and development which depend on a protein-rich diet (Seress and Liker 2015). A number of studies have demonstrated the unsuitability of an urban diet for nestlings, with some indicating that even granivorous birds prefer to feed nestlings a high-protein diet of insects, specifically in early developmental stages (Kalmback 1940; Mueller 1986). Importantly, experimentally manipulated nestling diets including higher fat or carbohydrate content and lower protein content have been linked with decreased nestling growth and body condition, indicating nutrient rather than calorie deficiencies (Newhouse et al. 2008; Heiss et al. 2009; Pollock et al. 2017). Thus, in our study, as nestlings experienced more HHP days, they ingested less protein-rich food and more carbohydrates, likely leading to a deficiency in required nutrients resulting in impaired growth. Other stress-related effects of increased foot traffic, humans approaching nests and a potentially associated increase in nest defence behaviour (i.e. aggression of breeding starlings towards humans) on HHP days may also impact nestling growth and development and could result in a similar pattern to the one we observed. However, our results point to a diet- rather than disturbance-related mechanism because provisioning rates to nestlings remained constant on HHP and LHP days and only diet makeup changed. To clearly define this mechanism, experimental supplementation should be performed to test the hypothesis that food quality is the main determinant of nestling growth and development in this system. In addition, bomb calorimetry or another similar method could provide supplemental data on the nutritional value of the food nestlings are receiving and perhaps even their body composition (especially if non-invasive methods can be found, e.g. TOBEC measurements *sensu* Castro et al. (1990)).

Reduced nestling growth in broods exposed to more HHP days and associated low-quality anthropogenic food in our study system may have a range of implications for nestlings’ long-term fitness. Nutritional deficits in early nestling developmental stages have been shown to negatively influence health traits such as fatty acid profiles (Toledo et al. 2016), carotenoid-based colouration (Sumasgutner et al. 2018) and plasma cholesterol levels (Gavett and Wakeley 1986). Furthermore, urbanisation can alter defence physiology (immune function) in developing birds (Nwaogu et al. under review), with a number of observed longer-term negative impacts on fecundity and survival (reviewed in Metcalfe and Monaghan (2001)). For example, an urban diet was linked to telomere shortening in Great Tits (Salmón et al. 2016)—an indication of cellular senescence—and selective disappearance of individuals with shorter telomeres was observed (Salmón et al. 2017). In addition, smaller size or body condition prior to fledging has strongly predicted later recruitment as a breeding adult in many bird species (Schwagmeyer and Mock 2008; Sumasgutner et al. 2016). Thus, the quality of food provisioned to nestlings is extremely important, as early nutritional conditions heavily influence both their development and subsequent survival (Seress and Liker 2015).

The nestlings in our study that were exposed to more HHP days are likely to be disadvantaged in the long term as their smaller size may indicate nutritional shortfalls in their diet. Long-term monitoring is required to assess whether the recruitment rates of nestlings exposed to large numbers of HHP days are lower than those of their conspecifics who experienced more LHP days and received a higher-quality diet as a result. Indeed, one aspect worth considering is the potential benefit of having a lower wing loading at fledging. Lighter birds may be able to more effectively move and avoid predators in the vulnerable days and weeks following fledging, especially if some urban adapted predators are specialising on adult avian prey. This has been previously documented, where predators living in urban environments appear to prey switch from juvenile birds in nests (common prey items in rural environments) to free-flying, larger-bodied birds (Stracey 2011; Malone et al. 2017). In this case, the drawbacks associated with potentially poorer nutrition during the nestling phase resulting in a lighter weight (and potentially compromised body condition) could be outweighed by the potential increased chances of survival associated with lighter wing loading at fledging. In our study system, potential avian predators of starlings are the black sparrowhawk (*Accipiter melanoleucus*) and peregrine falcon (*Falco peregrinus*), both of which have healthy populations living in and around the study area (Rose et al. 2017; Sumasgutner et al. 2020). There is no evidence as yet to suggest these species are preferentially predating on larger-bodied adult starlings, but further research could test whether lower wing loading at fledging may be under positive selection in this urban environment in comparison to rural starling populations.

While most work in avian urban ecology has drawn comparisons between urban and rural populations within species (Chamberlain et al. 2009), we studied individual birds from a single population experiencing pulsed anthropogenic food availability—an approach which largely eliminates several potentially confounding factors such as location and individual bird identity. To add to our understanding of this system, further work could include long-term post-fledging survival tracking of these starlings, and compare these results, as well as the other results generated in this study, with those of rural nests. This may also widen our understanding of urbanisation as a potential evolutionary driver of adaptation in this context—a topic receiving increasing attention globally (Johnson and Munshi-South 2017).

Our study is one of the first to closely examine how urban birds respond behaviourally to fluctuating anthropogenic food supplies over a short timescale, while identifying a potential mechanism behind the widely observed trend of poor developmental parameters in urban nestlings (Chamberlain et al. 2009). That breeding adult starlings seem to benefit from increased anthropogenic food consumption in our study while their nestlings apparently suffer during development, raises the possibility that this urban environment is operating as an ecological trap (Schlaepfer et al. 2002; Battin 2004; Robertson and Hutto 2006; Hale and Swearer 2016). While further work would be needed to explore this possibility, we have shown that—although largely overlooked—the impacts of short-term fluctuations in availability of anthropogenic food in urban environments can be profound, both for breeding adults themselves and for the outcomes of their breeding attempts.

## Data Availability

The datasets generated and analysed during the current study are available as electronic material on UCT’s ZivaHub: 10.25375/uct.16610671.

## Code availability

We have used standardised modelling so no code is provided.
